# Prevalence and associated factors of breast cancer screening among nuns in the Catholic Archdiocese of Nairobi

**DOI:** 10.11604/pamj.2023.44.117.38005

**Published:** 2023-03-03

**Authors:** Alfena Julie Joseph, Grace Mbuthia, Rosemary Kawira

**Affiliations:** 1 Department of Community Health Nursing, Jomo Kenyatta University of Agriculture and Technology, Nairobi, Kenya

**Keywords:** Breast cancer, screening, nuns, cancer screening knowledge

## Abstract

**Introduction:**

breast cancer is a significant global challenge. The risk of breast cancer among nuns is high mainly due to the basis of nulliparity. Among the effective approaches to addressing breast cancer is early screening. However, there are concerns over the uptake of screening across all populations, including nuns. The objective of the study is to determine the prevalence and the associated factors of breast cancer screening among nuns in the Catholic Archdiocese of Nairobi.

**Methods:**

this research used the analytical cross-sectional design. A total of 384 nuns in the Catholic Archdiocese of Nairobi were recruited using a stratified sampling. Structured questionnaires were used to collect data. Chi-square and binary logistic regression were used to determine association between social-demographic factors and breast cancer screening. Statistical package for social sciences (SPSS version 26) was used for analysis. The level of significance was investigated at p<0.05.

**Results:**

our findings revealed that the prevalence of breast cancer screening among nuns was 30.7%. The findings established that those who had knowledge on breast cancer screening (AOR=25.52, 95%CI: 8.87 - 73.45, p<0.001) and those who had congregational financial support (AOR=1.97, 95%CI: 1.68 - 5.74, p=0.021) were more likely to undergo breast cancer screening. Those who had hospital check-up for in more than six months prior to the study (AOR=0.001, 95%CI: 0.000 - 0.008, p<0.001) and those who never had a hospital check-up, (AOR=0.001, 95%CI: 0.000 - 0.006, p<0.001) were less likely to undergo breast cancer screening.

**Conclusion:**

the findings have shown low uptake of breast cancer screening amongst nuns in the Catholic Archdiocese in Nairobi. Knowledge on breast cancer screening access to congregational support and hospital check-up have been identified as key factors associated with breast cancer screening. Therefore, there is a need to create adequate awareness be created and the Catholic reverend sisters educated through aggressive education campaign programs so as to eliminate misconceptions relating to the topic. Also, to integrate free access to screening services in the government health institutions for nuns.

## Introduction

Breast cancer remains a global challenge. According to the World Health Organisation (WHO), approximately 2.3 million women were diagnosed with the condition in 2020, and the global number of deaths was about 685,000. By the end of 2020, the number of women diagnosed and living with breast cancer was 7.8 million [1]. These statistics reflect on the prevalence of the condition, and WHO notes that it is the most prevalent type of cancer. Another core factor describing the impacts of the condition is that among all types of cancer, breast cancer has the highest number of lost disability-adjusted life years. Breast cancer also occurs in every nation among women of all ages after puberty, and the prevalence increases with age. The prevalence leads to the cause of cancer-based mortality and morbidity in sub-Saharan Africa (SSA) [2].

In Kenya, the National Cancer Screening Guidelines, in collaboration with the National Cancer Control Strategy (2017-2022), focuses on early cancer screening and prevention [3]. This reflects on the country´s strategies to respond to the rising cases of the condition. Among the core consideration factors is increasing the screening, which informs early treatment. According to Antabe *et al*. (2020), the prevalence of breast cancer in Kenya is 34 in 100,000 [4]. These figures are obtained from the 2015 Kenya Stepwise Survey of the Non-Communicable Disease Risk Factors Report. The prevalence amounts to 23% of all cancer cases [5].

The changes and improvements in mortality and morbidity of the condition commenced in the 1980s due to education programs and innovative treatment approaches to eradicate the condition. Among them, breast cancer screening (BCS) is an effective measure. As described by the Centre for Disease Control and Prevention (CDC) (2020), BCS entails checking the woman´s breasts for any symptoms or signs of breast cancer [6]. Every individual should be informed about the BCS approaches available for them. This information is essential in making informed decisions regarding the screening process and whether it is suitable for the person. This reflects on the shared decision-making process and person-centred care model. Breast cancer screening does not treat or prevent the condition. However, as espoused by Bevers *et al*. (2018), BCS plays a crucial role in identifying the signs of the condition, prompting for an early and effective treatment plan [7].

There is insufficient information regarding BCS among nuns in Kenya. The data available shows a low uptake of screening among all communities. A significant challenge is the high prevalence of breast cancer in Kenya among all communities. The rate of prevalence of breast cancer among nuns globally is high [8]. However, the uptake of BCS among African societies remains low [9]. There is little information regarding the rate of BCS among nuns in Kenya [4], which is the foundation of this study. It is, therefore, essential to determine the prevalence and socio-economic factors influencing breast cancer screening among nuns in the Catholic Archdiocese of Nairobi.

## Methods

**Specific objectives:** to determine the prevalence of breast cancer screening among the nuns of reproductive age in the Catholic Archdiocese of Nairobi. To determine the socio-economic factors associated with breast cancer screening among nuns in the Catholic Archdiocese of Nairobi.

**Study design:** the analytical cross-sectional was conducted with both quantitative and qualitative data approaches. The design was applied to determine the prevalence of breast cancer screening among nuns and the social, cultural, and economic factors influencing this prevalence. Breast cancer screening involved whether participants had undergone either of physical, clinical examination and mammography.

**Study setting and population:** the study was conducted at the Catholic Archdiocese of Nairobi. The study population comprised of nuns at the Catholic Archdiocese of Nairobi. Reproductive age (18-49) nuns in the Catholic Archdiocese of Nairobi were included in the study. Nuns with breast cancer and on treatment, and those above 49 years, were excluded. Those above 49 years were excluded because the study sought to assess breast cancer among nuns within the reproductive age. Those aged between 15 and 17 were not included in the study because there are no nuns within this age range. In investigating breast cancer screening, the study evaluated whether they had undergone any of the existing cancer screening approaches which include physical examination, clinical examination and mammography. The study was piloted from June 10 to 12, 2022. The study data was collected between 17^th^ June, and 20^th^ July, 2022.

**Sample size determination:** the sample size was calculated using the Fischer formula:


$$n=\frac{z^{2}P\left(1-P\right)}{e^{2}}$$

Where n is the desirable sample size, z is the z-score, p is the standard deviation, and e is the confidence interval.

In this research, the confidence interval was 95%, which has a z-score of 1.96 was used. A confidence interval of ±5% and a standard deviation of 0.5 were selected. The P of 0.5 was selected since the prevalence of breast cancer screening among nuns in Kenya was unknown. Fifty percent was selected since there are no prevalence statistics about breast cancer screening among nuns in Kenya. Substituting the values to the formula:


$$n=\frac{1.96^{2}\times 0.5\left(1-0.5\right)}{0.05^{2}}$$

Therefore, n = 384. The sample size for the research was selected as 384 nuns.

For the qualitative data, involving in-depth interviews (IDIs), the sample size was 12 nuns. The nuns were sampled from those who did not participate in the quantitative arm. According to Vasileiou *et al*. [10], the sample size in interviews and qualitative research ranges from 2 to 72. The low number of these individuals is attributed to the complexity in analysing the information. The IDIs was guided by data saturation.

**Sampling technique:** a multi-sampling technique was utilized to obtain data in this study. The data was sampled from the five orders. The total number of congregations from the five orders was 163 giving a total of 594 nuns. Proportionate sampling approach was used to ensure that there is equal representation of nuns sampled in each of the congregations. The sampling of nuns in each of the congregation, random sampling technique was used where each nun who met the inclusion criteria was recruited. In sampling each participant, the researcher folded papers written ‘yes´ and ‘no´. The ‘yes´ papers were equivalent to the sample size sought in each of the stratas. For qualitative data, a list all the nuns who had not participated in the quantitative arm was created using unique codes. The codes were randomized in Microsoft Excel where sampling was done until the saturation was attained.

**Variables:** the independent variables included in the study were both independent and dependent variables. The independent variables included demographic factors (age, education and occupation), socio-economic factors (knowledge on breast cancer screening, cultural beliefs, insurance cover, access to congregational finances), perception and experiences. The dependent variable was uptake of breast cancer screening.

### Data resource and measurement

**Data collection procedure:** those who were selected to participate in the study were issued with an informed consent form which those who agreed to participate in the study signed. Only those who signed consent forms were recruited into the study. This research used questionnaires to collect data. The interviewer-administered questionnaires with both open-ended and closed-ended questions were essential in collecting the data about the prevalence and the socio-economic factors. The questionnaires were self-administered. The questionnaires, administered by the researcher were collected physically within the Catholic Archdiocese of Nairobi. The questionnaires were presented to the respondents for filling and returned to the principal investigator for analysis. Data was collected over one month between 17^th^June, 2022 and 20^th^July, 2022 due to the high number of respondents. Nuns within the Catholic Archdiocese of Nairobi who participated in the study were asked whether they had ever had breast cancer screening which included physical, clinical examination and mammography.

**Data analysis:** this was done using SPSS version 26. Demographic characteristics of the study subjects were analyzed descriptively. Categorical data was analyzed using frequencies (n) and percentages (%) and represented in graphs and pie charts. Continuous data was analyzed using mean (SD) for normally distributed data or median (IQR) for skewed data. Chi-square, Fischer´s exact, students were used in bivariate analysis while binary logistic was used to conduct multivariate analysis. Fischer´s exact was used when cell frequency was less than five (n<5) while Chi-square test was used for cell frequency (n>5). Significance level required was <0.05. Odds ratio was used to show direction of association between the independent and dependent variables.

**Study limitation:** recall bias- some respondents failed to remember some important information being asked. This was addressed by limiting the study questions to a shorter period.

**Ethical consideration:** ethical clearance was given by Ethics Review Committee of Bara ton University (approval number: UEAB/ISERC/56/3/2022), National Council of Science and Technology and Innovation (NACOSTI) and Catholic Diocese of Nairobi secretariat. Confidentiality and privacy were observed.

## Results

A total of 384 questionnaires were distributed among study population. All the questionnaires were duly filled and returned for data analysis representing 100% response rate. The study also included 12 in-depth interviews.

**Social demographic characteristics of the respondents:** the findings established that 37.2% (143) of the respondents were aged between 30 to 39 years, 40.6% (156) had diploma level as their highest level of education. Further, 38% (146) of the respondents were teachers while 20.6% (n=79) were healthcare workers as shown in Table 1.

**Table 1:** social demographic characteristics of the respondents

Characteristics	Frequency	Percent
Mean age (mean ±SD)	34.8 ± 9.72	
**Age**		
18 - 29 years	130	33.9
30 - 39 years	143	37.2
40 - 49 years	69	18.0
≥50 years	42	10.9
**Highest education level**		
Secondary	80	20.8
Diploma	156	40.6
Undergraduate	95	24.7
Postgraduate	53	13.8
**Occupation**		
Accountant	32	8.3
Social worker	68	17.7
Catechist	5	1.3
Cateresses	12	3.1
Healthcare worker	79	20.6
Cleaner	9	2.3
Counsellor	16	4.2
Nutritionist	17	4.4
Teacher	146	38.0

**Prevalence of breast cancer screening among the nuns of reproductive age:** the results established that 30.7% (n=118) of the respondents had undergone breast cancer screening, 95%CI (26.2% - 35.6%).

**Awareness on breast cancer screening among respondents:** among those who had undergone cancer screening, 55.1% (65) had clinical palpation while 44.9% (53) had self-examination as method of screening. More than half, 58% (69) had breast cancer screening as a personal initiative while 6.7% (8) had screening because they had noticed a lump in their breast. In investigating the frequency of breast cancer screening among those who had screened, 41.5% (49) of the respondents had breast cancer screening at least once per year, 55.5% (66) had self-examination monthly. Among the respondents who had not had breast cancer screening, 47.4% (182) cited lack of awareness, 26.3% (101) fear. The findings also showed that 82.3% (316) of the respondents knew about the existence of breast cancer screening, 35.8% (113) of these respondents knew about breast cancer screening through learning institution, 26.9% (85) through social media. The results also show that 46.9% (180) of the respondents had received health education on breast cancer screening as shown in Table 2.

**Table 2:** awareness of breast cancer screening among respondents

Awareness and uptake	Frequency	Percent
**Method of screening (n=118)**		
Self-examination	53	44.9
Clinical palpation	65	55.1
**Purpose of breast cancer screening (n=118)**		
Personal initiative	69	58.0
Noticed a lump	8	6.7
Medical grounds	19	16.0
Advice from a health worker	22	19.3
**Frequency of breast cancer screening (n=118)**		
After every six months	22	18.6
At least once per year	49	41.5
Once in two years	47	39.8
**Frequency of self-examination (n=118)**		
Monthly	66	55.5
Every three months	22	18.5
Once a year	30	26.1
**Factors hindering breast cancer screening**		
Fear	101	26.3
Lack of finances	31	8.1
Lack of awareness	182	47.4
Peer pressure	61	15.9
Time	4	1.0
**Knowledge of breast cancer screening (n=384)**		
Yes	316	82.3
No	68	17.7
**Source of knowledge on breast cancer screening (n=316)**		
Learning institution	113	35.8
Social media	85	26.9
Community programs	63	19.9
Conferences and workshops	55	17.4
**Received health education on breast cancer screening (n=384)**		
Yes	180	46.9
No	204	53.1

**Socio-economic factors influencing breast cancer screening:** the findings established that slightly more than half, 53.4% (205) had never visited hospital for check-up. The factors hindering access to check up included fear 50.4% (135) and lack of resources 19.7% (52) among others. Among those who underwent breast cancer screening, 56.8% (67) stated that seeking breast cancer screening was a self-initiative while 28.8% (34) stated that their decision was influenced by the media. About, 29.9% (115) of the respondents had insurance cover, 14.1% (54) had access to congregational financial support. In assessing ease to access BCS among nuns, 77 (64.2%) had average ease in access to breast cancer screening while 1.3% (5) of the respondents had cultural and religious concerns as shown in Table 3.

**Table 3:** socio-economic factors influencing breast cancer screening among respondents

Factors	Frequency	Percent
**Visited hospital for a medical check-up (n=384)**		
Last one month	12	3.1
Last three month	15	3.9
Last six months	42	10.9
Last one year	34	8.9
More than one year ago	76	19.8
Never	205	53.4
**Factors hindering access to check up (n=266)**		
Time	32	12.1
Resources	52	19.7
Lack of support	47	17.8
Fear	135	50.4
**Influenced decision-making process for uptake (n=118)**		
Sibling	5	4.2
Self	67	56.8
Media	34	28.8
Friend	5	4.2
Referral	8	6.8
**Presence of insurance cover (n=384)**		
Yes	115	29.9
No	269	70.1
**Access to congregational financial support**		
Yes	54	14.1
No	330	85.9
**Rating on ease to access breast cancer screening**		
Low	36	30.0
Average	77	64.2
High	7	5.8
**Presence of any cultural and religious concerns that hinder breast cancer screening**		
Yes	5	1.3
No	379	98.7

**Factors associated with breast cancer screening among respondents** Pearson Chi-square and Fischer´s exact test were conducted to investigate factors associated with breast cancer screening as shown in Table 4. The findings revealed that occupation (χ^2^(3) = 14.524, p = 0.002), knowledge of breast cancer screening (χ^2^(1) = 21.185, p<0.002), receiving health education on breast cancer screening ((χ^2^(1) = 4.611, p = 0.032), hospital check-up in last six months (df = 5, p<0.001 and access to congregational financial support, ((χ^2^(1) = 14.762, p<0.001) were significantly associated with breast cancer screening.

**Table 4:** factors associated with breast cancer screening among respondents

Factors	BCS uptake				
	**Yes**	**No**	**Chi-square**	**Df**	**P-value**
**Age**					
18 - 29 years	34(28.8)	96(36.1)			
30 - 39 years	45(38.1)	98(36.8)	4.867^a^	3	0.182
40 - 49 years	28(23.7)	41(15.4)			
≥50 years	11(9.3)	31(11.7)			
**Education level**					
Secondary	21(17.8)	59(22.2)			
Diploma	49(41.5)	107(40.2)	1.185^a^	3	0.757
Undergraduate	32(27.1)	63(23.7)			
Postgraduate	16(13.6)	37(13.9)			
**Occupation**					
Accountant	9(7.6)	23(8.6)			
Social worker	31(26.3)	79(29.7)	14.524^a^	3	0.002
Healthcare worker	44(37.3)	52(19.5)			
Teacher	34(28.8)	112(42.1)			
**Knowledge of breast cancer screening**					
Yes	111(94.1)	196(73.7)	21.185^a^	1	p<0.001
No	7(5.9)	70(26.3)			
**Received health education on breast cancer screening**					
Yes	65(55.1)	115(43.2)	4.611^a^	1	0.032
No	53(44.9)	151(56.8)			
**Last hospital check-up**					
Last one month	10(8.5)	2(0.8)			
Last three month	14(11.9)	1(0.4)			
Last six months	19(16.1)	23(8.6)	^b^	5	p<0.001
Last one year	15(12.7)	19(7.1)			
More than one year ago	58(49.2)	18(6.8)			
Never	2(1.7)	203(76.3)			
**Have insurance cover**					
Yes	41(34.7)	74(27.8)	1.869^a^	1	0.172
No	77(65.3)	192(72.2)			
**Access to congregational financial support**					
Yes	33(28)	32(12)	14.762^a^	1	p<0.001
No	85(72)	234(88)			
**Presence of cultural beliefs and concerns**					
Yes	1(0.8)	4(1.5)	^b^	1	0.601
No	117(99.2)	262(98.5)			

BCS: breast cancer screening; ^a^: Chi-square test; ^b^: Fischer’s exact test; Df: degrees of freedom

**Binary logistic regression analysis of sociodemographic factors associated with BCS:** binary logistic regression analysis was conducted as shown in Table 5. Those who were accountants by occupation were 64% less likely to undertake breast cancer screening compared to those who were healthcare workers, (COR = 0.36, 95%CI: 0.21 - 0.63, p<0.001). Respondents who had knowledge about breast cancer screening were 5.66 times more likely to undertake breast cancer screening, (COR = 5.66, 95%CI: 2.52 - 12.74, p<0.001). Those who received health education on breast cancer screening were 1.6 times more likely to undertake breast cancer screening compared to those who had not received health education on breast cancer screening (COR = 1.61, 95%CI: 1.04 - 2.49, p=0.032). Those who had hospital check-up for more than six months were 0.006 times less likely to undertake breast cancer screening compared to those who had hospital check-up for less than past six months (COR=0.006, 95%CI: 0.001 - 0.026, p<0.001). Further those who had never had a hospital check-up were 0.005 times less likely to undertake breast cancer screening (COR=0.005, 95%CI: 0.001 - 0.021, p<0.001). Those who had congregational financial support were 2.8 times more likely to undertake breast cancer screening (COR = 2.84, 95%CI: 1.65 - 4.9, p<0.001).

**Table 5:** binary logistic regression of factors sociodemographic factors associated with breast cancer screening

			95% C.I.		
**Socio-demographic factors**	**S.E**.	**COR**	**Lower**	**Upper**	**P-value**
**Occupation**					
Healthcare worker		Ref			
Social worker	0.439	0.776	0.328	1.835	0.563
Teacher	0.289	0.774	0.439	1.362	0.374
Accountant	0.283	0.359	0.206	0.625	p<0.001
**Knowledge of breast cancer screening**					
Yes	0.414	5.663	2.517	12.744	p<0.001
No		Ref			
**Received health education on breast cancer screening**					
Yes	0.223	1.610	1.041	2.491	0.032
No		Ref			
**Last check-up**					
≤6 months		Ref			
>6 months	0.753	0.006	0.001	0.026	p<0.001
Never	0.739	0.005	0.001	0.021	p<0.001
**Access to congregational financial support**					
Yes	0.279	2.839	1.645	4.901	p<0.001
No		Ref			

S.E.: standard error; COR: crude odds ratio; Ref: reference category

**Multivariate analysis of factors associated with breast cancer screening:** the study also investigated independent factors associated with breast cancer screening. Variables that were significant under bivariate model were included in a multivariate model to control for any confounders as shown in Table 6. The findings established that those who had knowledge on breast cancer screening were 25.5 times more likely to undertake breast cancer screening (AOR =25.52, 95%CI: 8.87 - 73.45, p<0.001). Those who had hospital check-up for more than six months prior were 0.001 times less likely to undertake breast cancer screening compared to those who had hospital check-up for less than six months prior (AOR=0.001, 95%CI: 0.000 - 0.008, p<0.001). Also, those who had never had a hospital check-up were 0.001 times less likely to undertake breast cancer screening (AOR=0.001, 95%CI: 0.000 - 0.006, p<0.001). Those who had congregational financial support were 1.97 times more likely to undertake breast cancer screening (AOR = 1.97, 95%CI: 1.68 - 5.74, p=0.021).

**Table 6:** multivariate analysis of factors associated with breast cancer screening

			95% C.I.		
**Factors**	**S.E**.	**AOR**	**Lower**	**Upper**	**P-value**
**Occupation**					
Healthcare worker		Ref			
Social worker	1.152	0.203	0.021	1.947	0.167
Teacher	0.511	1.387	0.509	3.777	0.522
Accountant	0.624	0.305	0.090	1.037	0.057
**Knowledge of breast cancer screening**					
Yes	0.539	25.517	8.865	73.449	P<0.001
No		Ref			
**Received health education on breast cancer screening**					
Yes	0.453	1.835	0.756	4.457	0.180
No			Ref		
**Last check-up**					
Last six months		Ref			
>1 year	0.872	0.001	0.000	0.008	P<0.001
Never	0.858	0.001	0.000	0.006	P<0.001
**Access to congregational financial support**					
Yes	0.545	1.970	1.676	5.737	0.021
No		Ref			

S.E.: standard error; AOR: adjusted odds ratio; Ref: reference category

**Perceptions and lived experiences on breast cancer screening among nuns in the Catholic Archdiocese of Nairobi**

### Awareness on breast cancer screening

**Severity of breast cancer and predisposition of nuns to breast cancer:** the respondents demonstrated knowledge on the severity of breast cancer as cause of mortality as illustrated by one of the respondents who had this to say: *“breast cancer is a leading cause of female mortality globally”* IDI 4. In addition, a number of respondents were aware of the fact that nuns are more predisposed to breast cancer compared to other females as can be observed from the following responses: *“they told us that being a nun is one of the factors that can easily predispose you, so you are supposed to have regular check-ups”*IDI 3. *“To nuns, the cancer is common due to the null parity issue, which involves the reproduction activity”*IDI 4.

**Importance of screening for early detection and management of breast cancer:** all the respondents were aware on the need to screen for breast cancer for early detection. They emphasized on the importance of early detection of breast cancer for better outcomes in treatment and management of breast cancer as alluded to by the following responses: *“from what I hear from the media or when we go to hospital is that any cancer especially for breast cancer we try to go, if it is caught early, it can be treated, but if it is caught very late there will be a problem in treating”*IDI 7.

**Methods of breast cancer screening:** they were also knowledgeable on various methods of breast cancer screening including clinical breast examination, imaging - ultrasounds, mammograms as alluded to by the following respondents: *“during the scanning and clinical assessment, I have heard that the doctor will be looking at masses and lumps by feeling the breasts. This is also the case with the scanning where the doctor will look at the images and determine if there are any abnormalities. I also hear that the doctor can order more tests including a biopsy to determine the nature of the cells”*IDI 1.

**Experiences of breast cancer screening:** the study also sought to determine the lived experiences of breast cancer screening among nuns who had undergone breast cancer screening.

**Fear of the procedure:** despite the fact that the respondents understood the importance of breast cancer screening, they expressed fear of undergoing the procedure as can be noted from the responses below: *“I feared, I went once, I was unwell then the doctor said I should get a screening and they examined if there were any lumps or swelling in the breast that would suggest if I had the cancer”*IDI 8. *“I was fearful, now when we being taught about cancer, I thought maybe what if I do this and it turns out to be positive, maybe I get a lump but I thank God it was not positive”*IDI 6.

**Preference of self-breast exam as opposed to clinical palpation:** some respondents prefer to perform self-breast examination as opposed to clinical examination of the breast for different reasons. This includes lack of time and expenses associated with clinical examination as illustrated: *“I find it easy doing it on myself because it doesn´t take so long”*IDI 10. *“Because we don´t have an insurance, some of us cannot be able to go for breast cancer screening because it is expensive”*IDI 11.

**Challenges hindering screening:** accordingly, there was low uptake of breast cancer screening which they attributed to lack of funds as well as time to go for the screening. Similarly, lack of information among most nuns hindered BCS among the nuns. Some of the respondents had this to say: *“I would say lack of information because like myself it´s because of the training we once underwent that made me realize the importance of doing the screening”*IDI 12. *“Mostly us nuns we don´t create the time maybe wherever you are, you are being sent somewhere by the congregation you don´t have much time but you can go for the screenings”* IDI 9.

## Discussion

The present study investigated the breast cancer screening among nuns in the Catholic Archdiocese of Nairobi. The findings from the present study revealed that the average age of the participants was 34.8 years. These findings contrast those from a study conducted in Uganda which revealed that 57.7% were aged between 41 to 60 years [11]. Another study conducted by Thiel *et al*. (2008) in a study conducted in United States where the average age of age of participants was 64 years [12]. This could be attributed to the difference in target population. In our present study, the target population comprised of nuns at the Catholic Archdiocese of Nairobi while in their studies, the population consisted of only nuns who came for digital mammography at the hospital.

The present study also established that majority of the respondents had tertiary level education with 40.6% having diploma qualification, 24.7% having undergraduate qualification while 13.85 had post graduate qualification. These findings are comparable to a study in Nigeria by Onyawoi (2014) which revealed that 52% of the respondents had tertiary level education while 20.1% had postgraduate level education [13]. Catholic nuns dedicate their lives to service. They work tirelessly to improve the conditions of their congregations and communities. They are also addressing many of the serious issues that plague the African continent. African nuns are also deeply trusted members of their communities. They are leading and serving where the needs are the greatest services sectors such as in schools, in healthcare facilities, and in human service, environmental and economic projects across the continent. These settings require that they gain addition al skills and knowledge through tertiary and post graduate education.

Our findings revealed that a third of the nuns had breast cancer screening which is relatively lower considering higher risk among nuns. The low breast cancer screening practice among nuns has been revealed in other studies [13,14]. Allen *et al*. (2014) in a study conducted in among Catholic Latinos revealed that breast cancer screening uptake was low at 24%. The barriers to screening among Latinos has been investigated which include lack of health insurance, concerns about cost, perceived discrimination, inadequate awareness and lack of provider recommendations. The role of religious factors in cancer screening behaviours may be especially pertinent to Latinos in the United States who, as a whole, express high levels of religious devotion and religious service attendance [14]. Therefore, understanding the role of religious beliefs, traditions, bodies of doctrine, and ministry in shaping health beliefs and behaviours is of critical importance. Onyawoi (2014) in a study conducted in Nigeria investigating cancer screening among catholic nuns found that prevalence of breast cancer screening was 27% [13]. Breast cancer screening in general population has been slightly higher compared to the prevalence among nuns. This is evident in a qualitative study conducted in Kenya investigating breast cancer screening in Coastal Kenya established that prevalence of breast cancer screening was 40 percent [4]. This is mainly attributed to less restrictions especially in terms of finances required to undergo breast cancer screening or presence of medical camps across different settings which are uncommon for nuns [4,15].

The current study revealed that knowledge on breast cancer screening was significantly associated with breast cancer screening among nuns. Those who had knowledge on breast cancer screening were 26 times more likely to have breast cancer screening. These findings are comparable to Abeje *et al*. (2019) in Ethiopia who revealed that women who had knowledge about breast cancer screening were more likely to have breast cancer screening where 97 percent among those who had breast cancer knowledge had undergone breast cancer screening [16]. Similarly, a study conducted in Coastal Kenya, revealed that knowledge about breast cancer screening was associated with increased breast cancer seeking behaviour [4]. In Nigeria, a study conducted by Madu and Nkem (2014) revealed that having low knowledge level was associated with poor breast cancer screening behaviour [17]. Education and continuous sensitization to the signs and symptoms of breast cancer, the fact that early detection increases the chances for cure, and the options for screening, diagnosis and treatment will be key to empowering people with accurate knowledge and dispelling some of the myths about breast cancer, which will encourage women to seek breast health services in a timely manner.

Our present findings also revealed that, having hospital check-up was associated with breast cancer screening. Having a hospital check is significantly associated with positive health seeking behaviour. In our present study, those who had hospital check-up of more than six months prior to the study had a reduced chance of seeking breast cancer screening. These findings were comparable to past studies [11,18] Orindi (2016) in a study conducted in Kisii, Kenya, revealed that those who had poor health seeking behaviour were less likely to undergo breast cancer screening [18]. Health seeking behaviour in this context involves having hospital check which help in identification of common illnesses. Similarly, Basaza *et al*. (2022) in a study in Uganda revealed that those who had hospital check-up had a higher likelihood of breast cancer screening [11].

The current study revealed that respondents who received congregational financial support were more likely to have breast cancer screening. These findings compare with those from Orindi (2016) in a study done in Kenya which stated that low socio-economic status was associated with low breast cancer screening [18]. Only those who are able to afford breast cancer screening especially clinical examination and mammography. Congregational financial support is one of the ways that nuns have access to finances and thus those who have access to these funds are able to pay for their cancer screening.

## Conclusion

The findings from the current study have showed that breast cancer screening among nuns was low at 30.7%. The findings revealed that occupation of nuns was associated with breast cancer screening. Those who worked as accountants were less likely to undergo breast cancer screening. The low breast cancer screening was associated with knowledge of breast cancer screening, having hospital check-up of less than six months and having access to congregational financial support. Therefore, there is need to create adequate awareness be created and the Catholic reverend sisters educated through aggressive education campaign programs so as to eliminate misconceptions relating to the breast cancer screening. In addition, there is need to provide health insurance cover for nuns to alleviate the challenge of financial support in seeking breast cancer screening. Other recommendations include to encourage regular check-up among nuns to improve on breast cancer screening. Provision of breast cancer screening camps in different orders to improve health check-up and increased understanding of their overall wellbeing is essential.

### What is known about this topic

- *There is no known magnitude of breast cancer screening among nuns in Kenya despite high vulnerability to breast cancer*.

### What this study adds

- *The study has provided the prevalence of breast cancer screening among nuns as well as associated factors*.
